# Vibration-induced illusion of movement is hindered by acute stroke but mostly by aging: a cross-sectional study

**DOI:** 10.1007/s40520-025-03247-6

**Published:** 2025-12-01

**Authors:** Brieuc Léger, Pascal Auzou, Élodie Fourdrinoy, Mathilde Sarrazin, Sylvine Celot, Céline Gay, Barbara de Dieuleveult, Clara Cohen, Stéphane Perrey, Canan Özsancak

**Affiliations:** 1 Service de Neurologie et Unité Neurovasculaire, Centre Hospitalier Universitaire d’Orléans, 14 Avenue de l’Hôpital, Orléans Cedex 2, 45067 France; 2 EuroMov Digital Health in Motion, Univ Montpellier, IMT Mines Alès, Montpellier, France; 3 Li 2 RSO, Université d’Orléans, 14 Avenue de l’Hôpital, Orléans Cedex 2, 45067 France; 4 Service de Neuroradiologie, Centre Hospitalier Universitaire d’Orléans, 14 Avenue de l’Hôpital, Orléans Cedex 2, 45067 France

**Keywords:** Vibration, Illusion, Movement, Aging, Stroke, Proprioception

## Abstract

The proprioceptive and visual systems play a major role in daily tasks by providing continuous feedback to the central nervous system (CNS) for coordinating movements. However, it remains unclear to what extent alterations in the proprioceptive system and CNS affect vibration-induced illusion of movement (VIM) with age and after a stroke. To address this, 29 young (26 ± 7 years), 30 older (63 ± 8 years), and 26 stroke participants (68 ± 12 years) with left arm impairment, all right-handed, received triceps brachii tendon vibration with or without visual feedback of the vibrated arm (with/without vision), as it can modulate the illusion of movement. Vibrations were applied bilaterally in healthy participants and on the impaired left arm in stroke individuals. The illusion was quantified using the Standardized Kinesthetic Illusion Procedure (SKIP) ordinal scale, which evaluates the clearness and the direction of the movement, resulting in a total score on four. While young and older adults achieved higher scores without vision, acute stroke participants did not (**Young**_**left**_: µ_(without/with)_ = 2.62/0.86, *p* < 0.001, **Young**_**right**_: µ_(without/with)_ = 2.35/0.69, *p* < 0.001; **Older**_**left**_: µ_(without/with)_ = 1.52/0.63, *p* < 0.001, **Older**_**right**_: µ_(without/with)_ = 1.03/0.50, *p* < 0.01; **Stroke**: µ_(without/with)_ = 0.85/0.62, *p* = 0.23). Moreover, young participants reported a stronger illusion than both older and acute stroke participants, and older participants reported a stronger illusion than acute stroke participants. Altogether, these findings suggest that aging alters VIM response, with acute stroke exacerbating this impairment. Finally, in acute stroke participants, a significant negative correlation between age and SKIP total score without vision was observed, highlighting the considerable impact of aging even within a pathological condition.

Clinical Trial registration: NCT06218563–2024-01-12.

**Graphical abstract:**

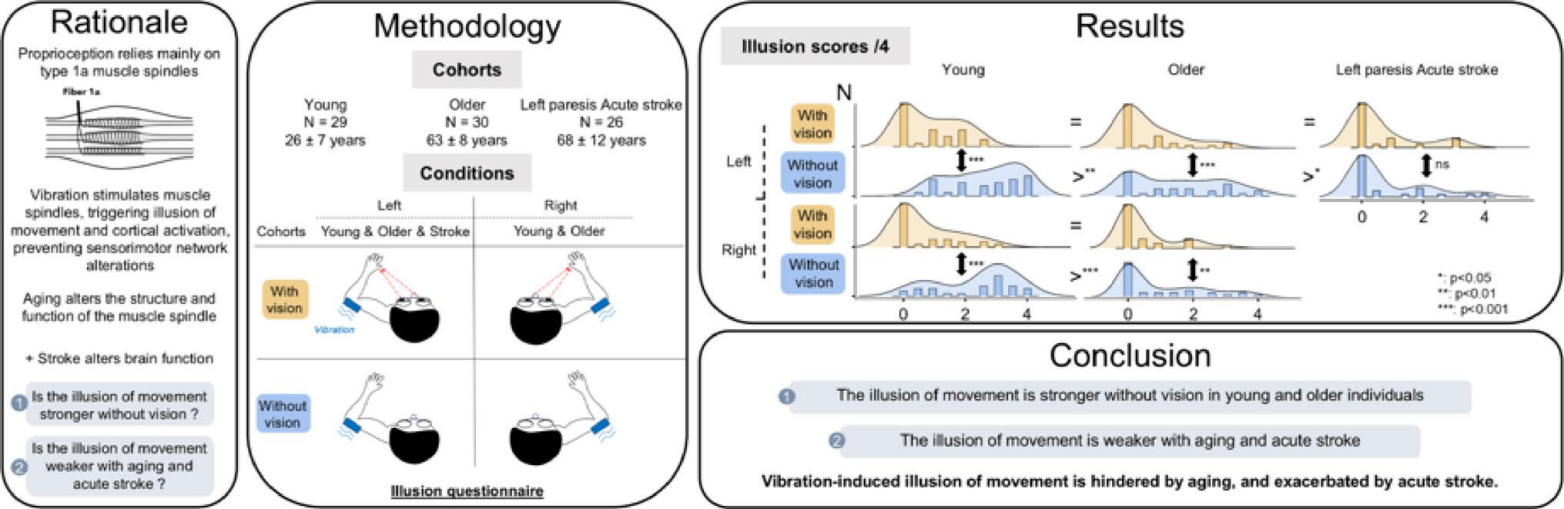

**Supplementary Information:**

The online version contains supplementary material available at 10.1007/s40520-025-03247-6.

## Introduction

In daily life, information from our body plays a crucial role, helping us to orient ourselves and adapt to sudden environmental changes. The perceived sensations of limb position and movement are made possible by specialized receptors called proprioceptors, which contribute to conscious body awareness [1]. In their review, Proske & Gandevia (2012) explain that proprioceptive afferents originate from various mechanoreceptors located in the skin, joints and muscles. Among these, the sensation of a stretched limb primarily arises from the activation of muscle spindle primary endings, which transmit signals via group 1a afferent fibers [1–3].

Experimentally, group 1a fibers can be optimally stimulated by muscle vibration at a frequency around 70–80 Hz [4–6]. Stimulating group 1a fibers through tendinous vibration generates a stretch signal from the muscle, which simultaneously produces a conscious sensation of movement in the direction of muscle elongation, commonly referred to as an illusion of movement [7]. However, this illusion can be disrupted by conflicting sensory feedback, such as visual input indicating that the limb is immobile. According to Guerraz et al. (2012), each sensory modality contributes to the final percept based on a weighting process, where more reliable sources of information are given greater weight. This suggests that vision, often deemed highly reliable, can dominate proprioceptive input and reduce or even suppress the illusion when visual feedback contradicts the expected movement [8, 9]. But other factors beyond sensory inputs can also influence the illusion. For instance, some studies have reported that the vividness of the illusion can vary between arms, and that gender and handedness influence the perceived velocity and extent of the movement [10, 11]. In addition, while aging has become a major public health concern [12], its impact on vibration-induced illusion of movement (VIM) and their neurophysiological correlates remains poorly understood. Aging is associated with structural and functional deterioration of muscle spindles [13], which in turn alters corticospinal responsiveness [14–17]. Investigating VIM in older adults could therefore provide valuable insights into age-related changes in sensorimotor integration and cortical plasticity, supporting the development of tailored rehabilitation strategies and preventive interventions.

VIM induces cerebral activations similar to those associated with limb movement in the sensorimotor and frontoparietal areas, and thus holds potential as a neuromodulation therapy [18–23]. It may even prevent sensorimotor network alterations caused by limb immobilization [24]. This is particularly relevant in clinical contexts such as stroke rehabilitation, where proprioceptive impairments [25], disturbances in body ownership [26], and motor deficits may occur [27].

To our knowledge, VIM has been studied in chronic [28–30] and subacute stroke [31, 32], but not in acute stroke. Previous studies have shown that VIM can effectively induce the illusion [29, 30] and enhance immediate reaching performance [28], while also promoting motor function recovery [31] and revealing cerebral responsiveness [33]. However, brain reorganization in acute stroke is complex and unclear [34], and the cerebral recovery appears slow. Earlier reports showed reduced VIM clarity on the paretic side of chronic stroke patients [29] and in a case study three weeks post-stroke [32]. Together, these findings suggest that individuals with acute stroke may struggle to perceive the VIM. Considering the aging of the world’s population [35], the increasing prevalence of stroke with age [36], and the high frequency of upper limb proprioceptive impairment after stroke (43–63% depending on the affected limb) [25], there is a clear need to further investigate VIM in older adults and in individuals with acute stroke.

Therefore, the aim of this study was to investigate how VIM is affected by aging and acute stroke, with or without visual feedback when applied to the vibrated left or right arm in healthy young and older adults, and to the paretic arm in acute stroke patients. Given that proprioceptive decline occurs with aging and that sensorimotor areas contribute to the generation of the illusion of movement, it was hypothesized that all participants would experience a stronger VIM without visual feedback of the vibrated arm, but that the illusion would be reduced in older adults and in acute stroke patients.

## Materials and methods

### Participants

A total of 30 young healthy participants aged 19–39 years (15 women, mean age: 25.97 ± 6.50 years), 30 older healthy participants aged 49–77 years (14 women, mean age: 63.23 ± 7.88 years) and 30 acute stroke participants (< 14 days post stroke) aged 45–85 years (14 women, mean age: 67.83 ± 11.19 years) participated in the study. All experiments were performed at the Neurology Department of the University Hospital of Orléans (France). Healthy participants were recruited between January 2024 and March 2024 through a flyer distribution campaign and word of mouth, and received a financial compensation of 50€ for their participation in the study. All participants were screened for right-handedness using the Edinburgh Handedness Inventory [37] and underwent the Thumb Localizing Test (TLT) [38], which respectively provide a laterality index (inclusion criteria: > 40) and three proprioceptive deficit scores. The TLT involves locating the tip of the thumb with the opposite hand while keeping the eyes closed, using three different arm positions for evaluation. The scoring ranges from 0 (normal) to 3 (severely impaired). For acute stroke participants, only the paretic side was evaluated, meaning they were required to locate the thumb of the paretic hand using their non-paretic hand. As Otaka et al. (2020) used the median TLT score to investigate correlations with quantitative assessments (robotic device), we similarly reported and used the median of the three scores in this paper.

Acute stroke participants were admitted to the neurology unit between January 2024 and June 2025. The inclusion criteria were as follows: patients had to be aged between 18 and 85 years, have experienced a first-ever ischemic or hemorrhagic stroke in the right hemisphere, and therapists had to note a left motor deficit in the upper limb. Furthermore, although not quantified, therapists were required to confirm the absence of significant cognitive impairments and aphasia. The participants were assessed using the NIHSS [39] upon admission to the unit and within 24-hours of the experiment. In this 24-hour period, motor ability and spatial neglect were also assessed using the following tests: the Fugl-Meyer Assessment for the Upper Extremity (FMA-UE) [40], the Bells Test [41] and the Line Bisection Test.

The study protocol was approved by the Institutional Review Board (CPP Sud-Méditerranée 1) on the 14 June 2023 and was registered under the number 2023-A01062-43 (Jardé law, RIPH2). It was also registered on clinicaltrials.gov under the identifier NCT06218563. All participants provided free and informed written consent in accordance with the Declaration of Helsinki. The study is reported according to the Strengthening the Reporting of Observational Studies in Epidemiology (STROBE) statement.

### Procedure

**Fig. 1:**
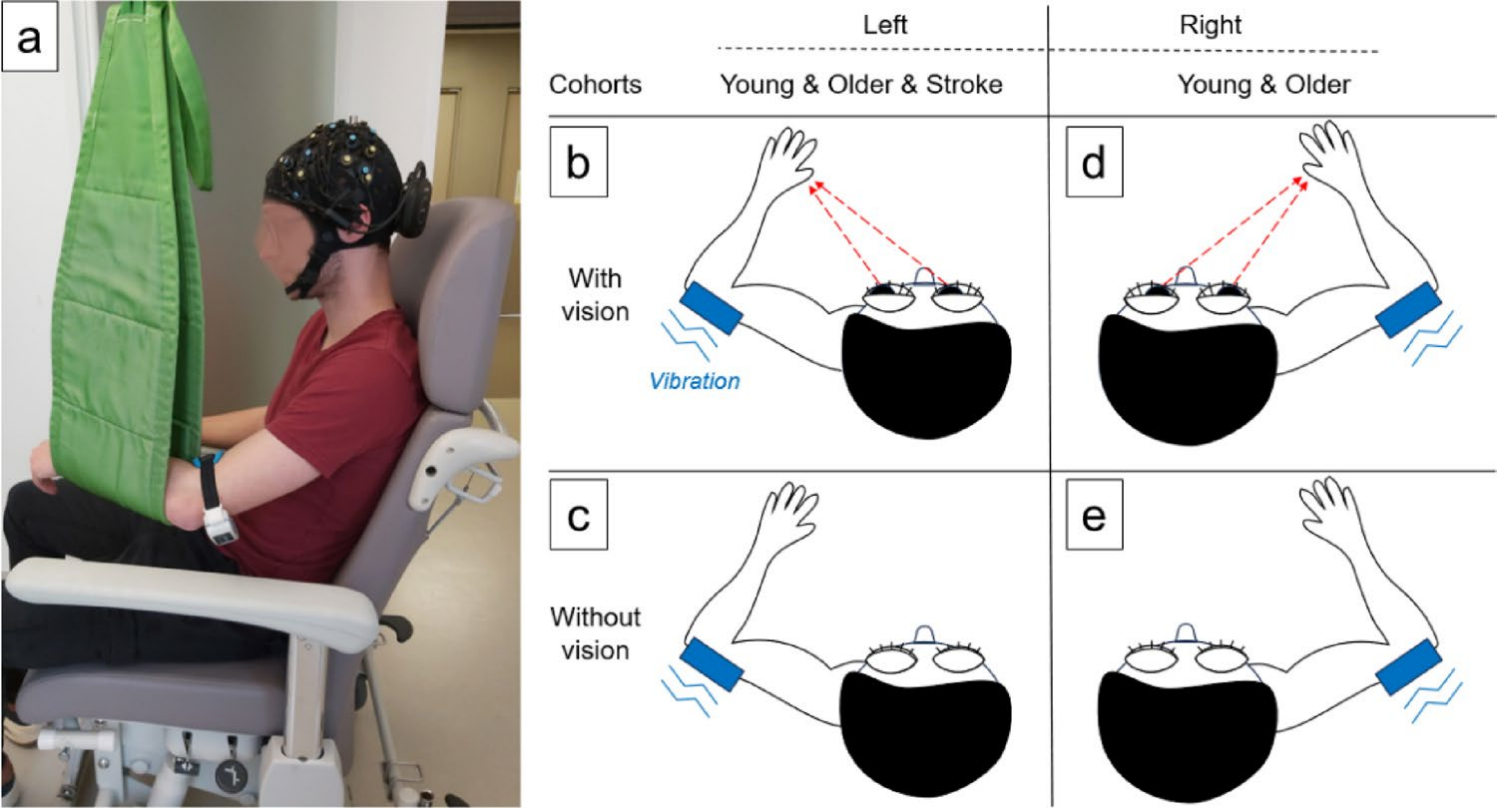
(**a**) Picture of the set-up in the quiet, dedicated room, showing a young participant seated in the chair. The movable green strap supports the left arm, with the vibrator placed on the distal tendon of the triceps brachii. Brain measurements were simultaneously collected using a near-infrared spectroscopy cap as part of a complementary analysis, which will be detailed in a later publication. (**b**-**c**-**d**-**e**) Schematic representation of the four modes of vibration. The vibrator is represented by the blue rectangle with blue lines showing the vibration. (**b**-**c**) Vibration of the left arm, performed on healthy young and older participants, and in acute stroke participants (paretic side). (**d**-**e**) Vibration of the right arm, only performed on healthy young and older participants. (**b**-**d**) With visual feedback of the vibrated arm, shown by the dotted red arrow pointing from the opened eyes to the hand. (**c**-**e**) Without visual feedback of the vibrated arm, shown by the closed eyes

The experiments were conducted in a quiet, dedicated room. Participants were seated comfortably in a chair, with their left or right arm gently placed in a support strap that left the distal triceps brachii tendon accessible (Fig. 1). The arm support was not completely fixed in order to avoid altering the sensation of movement [42]. To correctly place the vibrator device (Vibramoov Physio, Techno Concept, Mane, France), the participant’s arm was flexed in order to palpate the apex of the elbow as a reference point. The device was then placed a few centimeters above this, on the distal tendon of the triceps brachii. Participants were explicitly instructed to remain relaxed, in order to limit the tonic vibration response and favor the VIM [43], and to focus on the sensation of the elbow joint as they could not only feel the vibration, but also perceive an illusory arm movement. Participants received a point for reporting an arm flexion (the expected movement), but were not informed of the expected direction in order to avoid introducing bias. Finally, participants were told to keep looking at their hand in the visual feedback condition. Tendinous vibrations were applied at 80 Hz for 15 s with an amplitude ranging from 1 to 2 mm [5, 43]. Brain measurements were simultaneously collected using near-infrared spectroscopy as part of a complementary analysis, which will be detailed in a later publication.

In healthy participants, vibrations were applied to either the left or the right arm, with or without direct visual feedback of the vibrated arm, resulting in four distinct conditions: with or without vision, and with the vibration applied to either the left or right arm (Fig. 1). These conditions were chosen based on prior evidence suggesting that visual feedback can attenuate the VIM [8, 9]. Each condition consisted of six vibrations, performed in blocks of three and repeated twice, resulting in eight blocks of three vibrations, for a total of 24 vibrations (2 × 3 vibrations × vision × arm). The order of the conditions was counterbalanced within each group. As acute stroke participants might have struggled to maintain focus during a lengthy task, and to prevent excessive fatigue, the experiments were conducted only on the impaired left arm. This reduced the total number of vibrations to 12 (2 × 3 vibrations × vision). A resting period of 25–35 s was included before each vibration to prevent muscle conditioning and anticipation which could influence the sensation of movement [43].

To quantify VIM responses, we used the SKIP scale [29], which consists of a 4-point ordinal rating assessing of the perceived movement at the elbow joint (0 = no illusion perceived, 1 = vague and not precise, 2 = moderately clear and precise, 3 = perfectly clear and precise). An additional point was given if the perceived movement corresponded to an expected arm flexion, yielding a total possible score of 4. If participants had difficulty describing the direction of the perceived movement, they were invited to reproduce it using their non-vibrated arm. SKIP scores were reported after each block of three vibrations, yielding two scores per condition. The mean of these two scores was used for subsequent analysis. We only used the scoring component of the SKIP procedure, which has previously been shown to discriminate between VIM responses in healthy and chronic stroke individuals [29].

### Statistics

The sample size was determined based on previous studies on the VIM, which included between 15 and 31 participants and were able to detect significant effects [14–16, 29]. Although no formal power analysis was performed, we considered that including 30 participants per group would provide sufficient sensitivity to detect potential differences.

Statistical analysis was conducted using RStudio software (v2024.09.0 + 375) on R (v4.4.1) and the *tidyverse*,* rstatix* and *ppcor* packages [44–47]. Parametric tests were used, as they have been shown to be robust for ordinal scales, even when applied to non-normally distributed data [48].

To verify that the young group was indeed younger than the older group and the acute stroke group, and that latter two groups were of a comparable age, a one-way ANOVA was performed on the age groups, followed by unpaired t-tests with Bonferroni-Holm correction.

Then, two two-way mixed ANOVAs were performed to investigate the effects of vision (within-subject factor) and groups (between-subjects factor) on the left and right arms. All ANOVAs effect sizes were calculated using partial eta squares (pes) and interpreted as small (0.01–0.06), moderate (0.06–0.14) or large (> 0.14). As all ANOVAs results were significant, paired t-tests were conducted to examine the effect of vision within each group for both the left and right arms (five two-by-two comparisons). Additionally, four ANOVAs were used to evaluate group effect across visual and arm conditions. As only the ANOVAs performed without vision yielded significant results, unpaired t-tests with Bonferroni-Holm correction were used to compare each group without vision in both the left and right arms (four two-by-two comparisons). Effect sizes for all t-tests were assessed using Cohen’s d [49] and interpreted as small (0.20–0.50), moderate (0.50–0.80) or large (> 0.80).

Next, the occurrence of the illusion without vision (SKIP total score > 0) was compared between groups and arms using six two-by-two Pearson’s chi-squared tests, corrected for Yates’ continuity. Furthermore, to specifically assess VIM strength in participants who perceived the illusion, group differences were examined using a one-way ANOVA for the left arm, and an unpaired t-test for the right arm.

To examine the effect of aging on the VIM in acute stroke participants, we performed a partial Pearson correlation between age and SKIP total scores without vision. Confounding factors were controlled for by including FMA-UE scores, lesion volumes, NIHSS, TLT scores, days since stroke and spatial neglect scales in the model using the *pcor* function. Similarly, to assess the relationship between stroke-related clinical outcomes and VIM clarity, we computed partial Pearson correlations between clinical scales and SKIP total scores, while controlling for age and the same set of clinical variables. Effect sizes were interpreted as small (*r* = 0.10–0.30), moderate (*r* = 0.30–0.50) or large (*r* > 0.50) [49]. Additionally, simple Pearson correlations were conducted between age and SKIP total scores for the left and right arms of healthy older participants, who were within a comparable age range to the acute stroke participants.

## Results

### Demographic data and clinical data

One of the healthy young participants (a 24-year-old man) experienced a technical error resulting in data loss, reducing the analysis to 29 participants (15 women, mean age: 26.03 ± 6.61 years). Furthermore, four stroke participants were excluded from analysis: One due to loss of consciousness during the experiment, one because she was left-handed, one because she was highly somnolent during the experiment and one because she had a motor impersistence. Consequently, 26 participants with acute stroke aged 45–85 years (10 women, mean age: 67.69 ± 11.65 years) were included in the analysis. The characteristics of the analyzed participants are detailed in Table 1. Furthermore, acute stroke participants had their sensorimotor network impaired; for details, functional and anatomical localizations of lesions are provided in the Supplementary materials. One-way ANOVA revealed a significant difference in age groups (F_(2,82)_ = 189.99, *p* < 0.001). Unpaired t-tests indicated that the participants in the young age group were significantly younger than those in the older and acute stroke groups, while the latter two groups had comparable ages (**Young < Older**: *p* < 0.001, **Young < Stroke**: *p* < 0.001, **Older = Stroke**: *p* = 0.06).

**Table 1:** Characteristics of young, older and acute stroke participants

Characteristics	Young healthy	Older healthy	Acute stroke
Sample size (n)	29	30	26
Years of age mean (SD) (range)	26.03 (6.61) (19–39)	63.23 (7.88) (49–77)	67.69 (11.65) (45–85)
Gender (n female/n male)	15/14	14/16	10/16
Handedness Laterality Index (SD)	86.36 (14.31)	92.43 (11.44)	93.42 (10.58)
Thumb Localizing Test			
Left mean (SD)	0 (0)	0 (0)	0.54 (0.81)
Right mean (SD)	0 (0)	0 (0)	–
Lesion			
Volume (mL) (SD)			11.14 (18.94)
Cortical-subcortical/mixed	–	–	25/1
Nature (ischemic/hemorrhagic)	–	–	25/1
Hemisphere (n: left/right)	–	–	0/26
Days since stroke (mean) (SD) (range)	–	–	6.77 (2.85) (1–12)
NIHSS (mean)			
Admission (SD)	–	–	8.15 (4.52)
Experiment (SD)	–	–	5.00 (3.41)
FMA-UE score (SD)	–	–	42.35 (22.30)
Spatial Neglect			
Line bisection			
5 mm mean (SD)	–	–	1.28 (2.08)
20 mm mean (SD)	–	–	10.13 (16.12)
Bells test			
Total omissions (mean) (SD)	–	–	5.58 (7.66)
L-R omissions (mean) (SD)	–	–	2.19 (2.93)

### SKIP total score

**Fig. 2:**
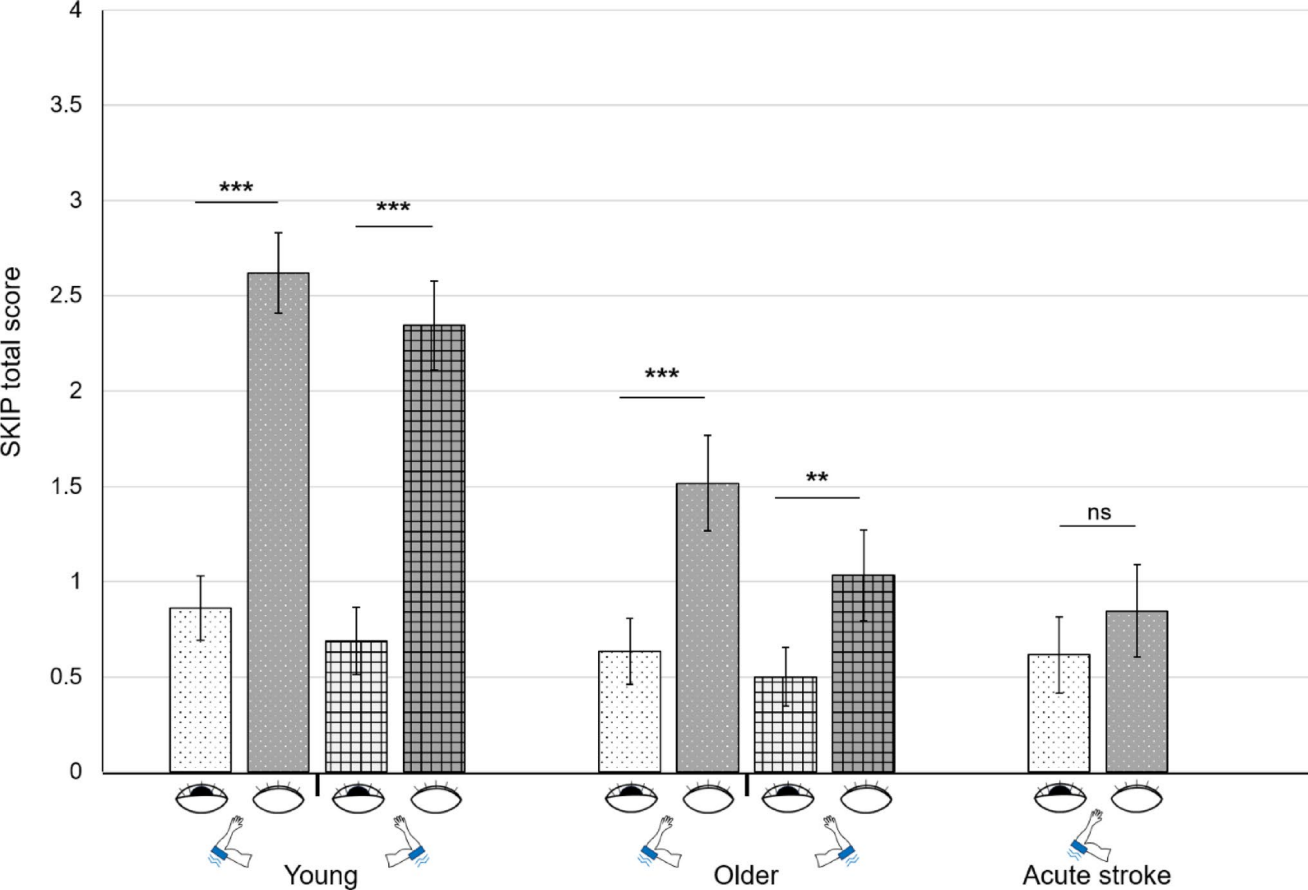
SKIP total scores (mean ± SE) for young, older and acute stroke participants. The first two dotted bars represent the left arm vibration conditions with (white bars with black dots) or without (grey bars with white dots) vision. The remaining two bars represent the right arm vibration conditions with (light squared) or without (dark squared) vision. Post-hoc paired t-tests: ns = non-significant; *p* < 0.01**; *p* < 0.001***

Firstly, the two-way mixed ANOVAs indicated significant results for both the left and right arms with an interaction effect of group × vision (**left**: F_(2,82)_ = 12.76, *p* < 0.001, pes = 0.24; **right**: F_(1,57)_ = 13.73, *p* < 0.001, pes = 0.19; Fig. 2), an effect of group (**left**: F_(2,82)_ = 7.96, *p* < 0.001, pes = 0.16; **right**: F_(1,57)_ = 9.36, *p* < 0.01, pes = 0.14; Fig. 2) and an effect of vision (**left**: F_(1,82)_ = 61.07, *p* < 0.001, pes = 0.43; **right**: F_(1,57)_ = 52.27, *p* < 0.001, pes = 0.48; Fig. 2). Post-hoc paired comparisons indicated higher total scores without than with vision in both young and older participants, and for the left and right arms (**Young**_**left**_: t = 7.21, *p* < 0.001, d = 1.34, **Young**_**right**_: t = 6.77, *p* < 0.001, d = 1.26; **Older**_**left**_: t = 4.55, *p* < 0.001, d = 0.83, **Older**_**right**_: t = 2.95, *p* < 0.01, d = 0.54; Fig. 2). However, no differences were found in stroke participants (t = 1.22, *p* = 0.23, d = 0.24; Fig. 2). In addition, post-hoc group comparisons indicated that young participants achieved higher total scores than older and acute stroke participants, and that older participants achieved higher scores than acute stroke participants without vision (**Young**_**left**_ **>Older**_**left**_: t = 3.35, *p* < 0.01, d = 0.87, **Young**_**right**_ **>Older**_**right**_: t = 3.93, *p* < 0.001, d = 1.02; **Young > Stroke**: t = 5.56; *p* < 0.001, d = 1.50; **Older > Stroke**: t = 1.96, *p* < 0.05, d = 0.51; Fig. 2).

Secondly, paired comparisons between the left and right arms of healthy participants revealed no significant differences in young and older participants without vision (**Young**: t = 1.00, *p* = 0.33, d = 0.19; **Older**: t = 1.98, *p* = 0.06, d = 0.36; Fig. 2).

### Influence of aging and acute stroke on illusion occurrence

**Fig. 3:**
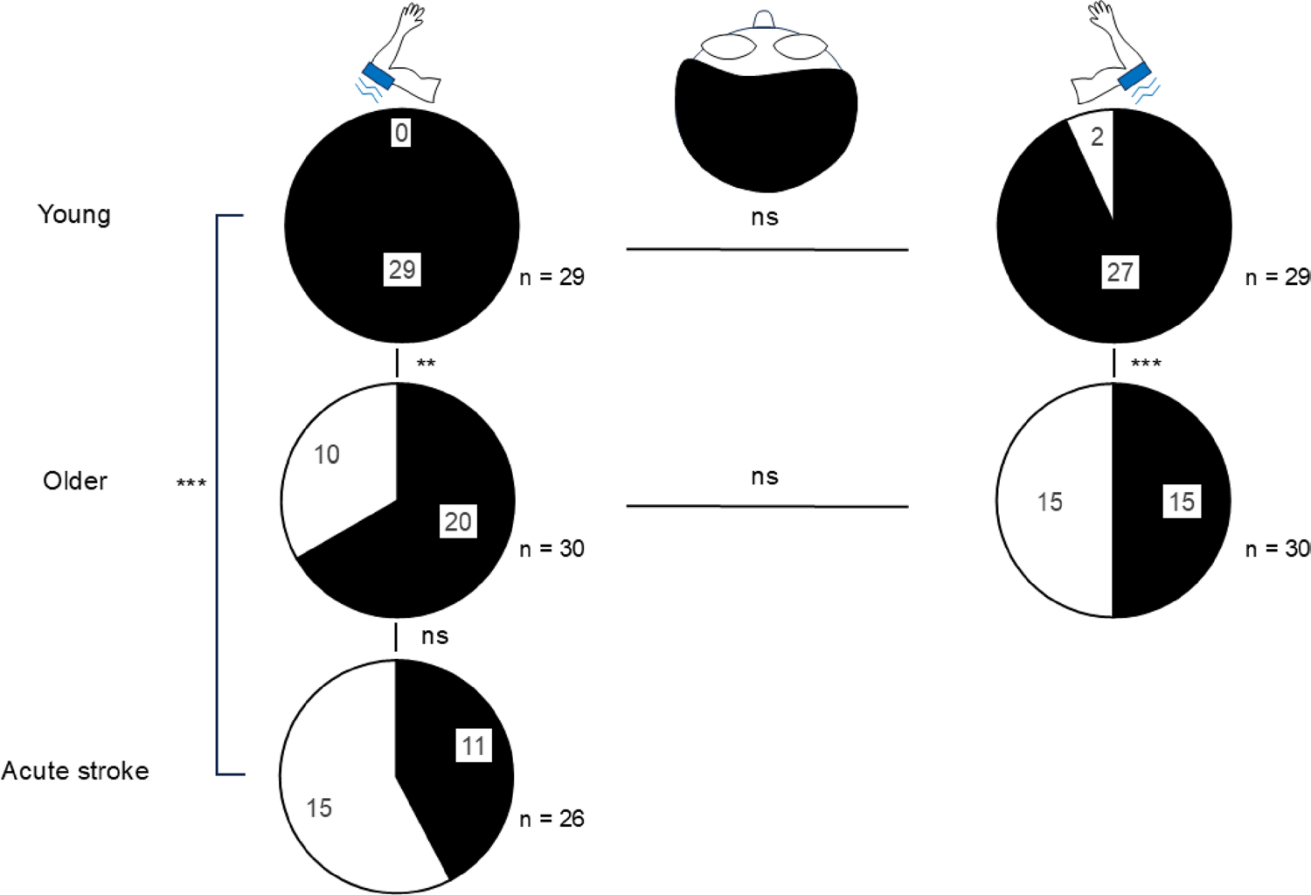
Illusion occurrences for young, older and acute stroke participants without vision (top, eyes closed). The vibrator is represented by the blue rectangle, and the blue lines show the vibration. On the left is the vibration of the left arm, performed on healthy young, older and acute stroke participants (paretic side). The right shows the vibration of the right arm, performed on healthy young and older participants. Black shows the number of participants who experienced the illusion of movement. White shows the number of participants who did not experience the illusion. Pearson’s chi-squared tests: ns = non-significant; *p* < 0.01**; *p* < 0.001***

The two-by-two Pearson’s Chi-squared tests (Fig. 3) revealed that a greater proportion of healthy young participants experienced an illusion of movement in both the left and right arms compared to older participants (**Young**_**left**_ **>Older**_**left**_: X² = 9.39, *p* < 0.01; **Young**_**right**_ **>Older**_**right**_: X² = 11.34, *p* < 0.001), and compared to acute stroke participants in the left arm (**Young > Stroke**: X² = 20.19, *p* < 0.001). However, no significant differences were found when comparing the proportion of older and stroke participants (**Older = Stroke**: X² = 2.43, *p* = 0.12). Finally, no significant differences were found for the illusion occurrences between the left and right arms of healthy young and older participants (**Young**: X² = 0.52, *p* = 0.47; **Older**: X² = 1.10, *p* = 0.29).

### Group comparisons of VIM strength

For the left arm, the one-way ANOVA showed no significant group differences among participants who perceived the illusion without vision (**Young**_**mean±SE**_: 2.62 ± 0.21, **Older**_**mean±SE**_: 2.28 ± 0.23, **Stroke**_**mean±SE**_: 2.00 ± 0.34) (F_(2,57)_ = 1.44, *p* = 0.25, pes = 0.05). Similarly, for the right arm, the unpaired t-test between young and older participants who perceived the illusion without vision was not significant (**Young**_**mean±SE**_: 2.52 ± 0.22, **Older**_**mean±SE**_: 2.07 ± 0.28) (t = 1.26, *p* = 0.22, d = 0.41).

### Associations of age and clinical outcomes with SKIP total scores

**Fig. 4:**
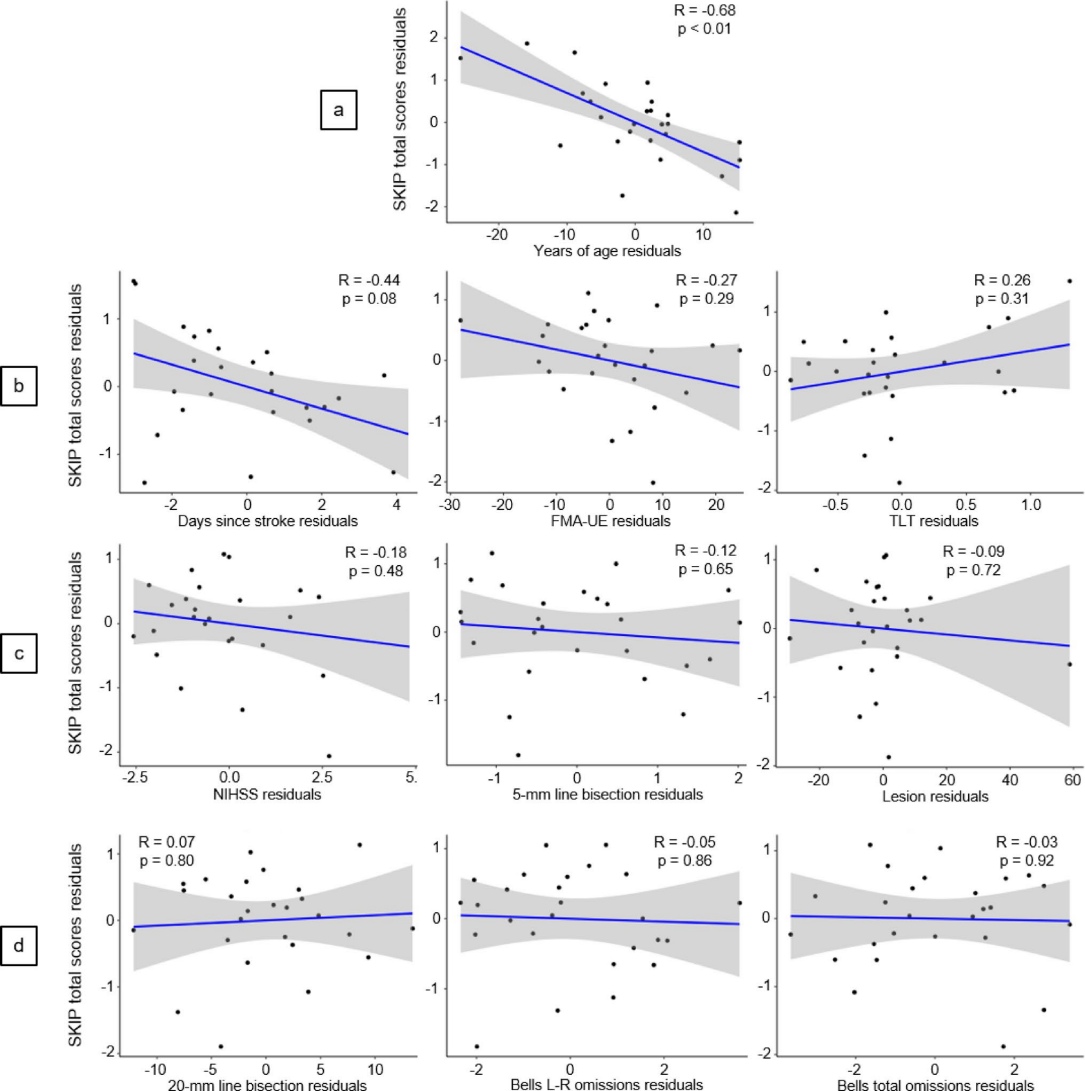
Partial Pearson correlations (grey areas represent the 95% confidence intervals) between SKIP total scores without vision and (**a**) age or (**b**–**d**) stroke-related clinical outcomes in acute stroke participants. For the correlation with age, residuals were computed after controlling for FMA-UE scores, lesion volumes, NIHSS, TLT scores, days since stroke, and spatial neglect scales. For correlations with clinical outcomes, residuals were computed after additionally controlling for age

No significant Pearson correlations were found between age and SKIP total scores in the left (*r* = -0.21, *p* = 0.26) and right arms (*r* = -0.05, *p* = 0.78) of healthy older individuals. However, a significant negative partial Pearson correlation was found between age and SKIP total scores when controlling for confounding factors such as FMA-UE scores, lesion volumes, NIHSS, TLT, days since stroke and spatial neglect scales in acute stroke individuals (*r* = -0.68, *p* < 0.01, Fig. 4a). Nevertheless, no significant correlations were found between any of the clinical outcomes and SKIP total scores in acute stroke participants (Fig. 4b-d).

## Discussion

We investigated how VIM is altered by aging and after an acute stroke, with or without vision in healthy young and older participants. In acute stroke participants, VIM was assessed from the left paretic arm. To this end, VIM was assessed and quantified using the SKIP scale [29].

Firstly, there were no significant differences in VIM responses between the left and right arms in healthy participants. Therefore, subsequent analyses focused only on vision conditions. Our results revealed that both young and older participants were able to effectively perceive the VIM. A significant effect of vision was observed: Healthy young and older participants had significantly higher SKIP total scores without vision than with vision. However, without vision, young participants exhibited significantly higher SKIP total scores and a greater proportion of VIM occurrences compared to older participants (Figs. 2 and 3), indicating an age-related decline in VIM response. In contrast, acute stroke participants did not show significant differences between vision conditions, suggesting that vision did not modulate VIM perception in this group. Additionally, although some acute stroke individuals were able to perceive the illusion, their SKIP total scores were significantly lower than those of healthy participants. However, when specifically assessing the VIM strength in participants who perceived the illusion without vision, no significant differences could be demonstrated between each group. Lastly, in acute stroke participants, SKIP total scores were independently affected by aging, but not by clinical outcomes (Fig. 4). The following sections discuss these different findings.

### VIM response related to laterality

Several authors have put forward a dynamic dominance hypothesis, positing that the dominant arm demonstrates greater efficiency and precision in dynamic tasks and that vision facilitates corrective adjustments [50, 51]. In contrast, the non-dominant arm would be particularly specialized in the control of steady-state position. This theory suggests that the dominant arm relies more on vision than the non-dominant arm for proprioceptive accuracy. Therefore, we might have expected higher VIM response in the left non-dominant arm of healthy participants.

However, our results showed no differences in VIM response between the left and right arms. This finding adds to the existing literature, in which no consensus has yet been reached. Previous studies have reported that individuals, whether right- or left-handed, experienced higher vividness of the illusion in the non-dominant compared to the dominant vibrated arm [11]. However, this result was only observed when participants received vibrations at the frequency that best elicited the VIM. Conversely, Adamo et al. (2012) reported that men experienced a faster VIM in the dominant arm compared to the non-dominant arm, a difference that was not observed in women. Additionally, it has been found that young right-handers experienced a faster VIM with their right arm than with their left arm, whereas no such difference was observed in older individuals [52]. Be that as it may, further specialized research is needed to better understand the effect of handedness on VIM responses.

### Vision effect on VIM responses

We demonstrated that healthy young and older individuals can experience the VIM, as evidenced by a significant increase in their SKIP total scores without vision compared to with vision.

This is in line with the pioneering study by Lackner & Taublieb (1984), which demonstrated that visual feedback of the vibrated arm attenuates the amplitude of the VIM. This is also consistent with the findings by Guerraz et al. (2012), who suggested that visual cues play a dominant role in the multisensory integration process. Together, these findings support a multisensory integration model in which VIM is influenced by visual input, either attenuating or enhancing the illusion [8, 9, 14, 42, 53, 54]. Our additional contribution underscores the importance of vision in the VIM and emphasizes that researchers should adapt their protocol depending on whether they want to attenuate or enhance VIM, or study the vibratory stimulation itself.

### Does aging have an effect on VIM discrimination and strength?

Older participants exhibited reduced VIM compared to young participants, as evidenced by lower SKIP total scores and fewer VIM occurrences, consistent with previous studies [14, 15]. However, unlike Chancel et al. (2018), we observed differences in the reported SKIP total score between young and older individuals, which relates to a concept similar to saliency in their study, although their participants were specifically asked to rate “vividness/clarity”. This discrepancy may be partly explained by the level of information provided to the participants. In their study, participants were exposed to the stimulus in a pre-test and in a training session, so they were aware of the expected movement, whereas in our study, participants did not know what was the expected direction of the movement. This may seem as negligible, but it has been shown that the level [55] and veracity of information [42] given to the participants can influence the perception of the VIM. Thus, in our study, older participants may have found it more difficult than young participants to experience a clear VIM, exacerbating the difference. Moreover, while Chancel et al. (2018) used vibration frequencies of 30 Hz and 60 Hz, we employed an 80 Hz vibration. It is generally acknowledged that the optimal frequencies to elicit VIM range between 70 and 80 Hz [4–6]. However, these frequencies might differ between young and older individuals. Chancel et al. (2018) reported significantly faster illusions at 60 Hz compared to 30 Hz in young participants, but this effect was not observed in older participants. A similar question was adressed by Landelle et al. (2018), demonstrating that VIM frequency discrimination threshold was higher in older participants than in young ones. These findings suggest that the VIM differences between older and young participants in our study were not due to unidentified confounding factors, but rather to the vibration frequency of 80 Hz used. However, when specifically assessing VIM strength in participants who experienced the illusion, no significant differences were found. This suggests that aging primarily influences VIM discriminative ability. Thus, an interaction between vibration frequency and aging might underlie changes in VIM. This may result from both peripheral and central deterioration associated with aging [17, 56, 57], including structural and functional alterations [13] of muscle spindles and cortical grey matter reduction [58]. Consistently, illusion discriminative ability has been shown to decline with age, more markedly when induced by vibration than by touch, suggesting a peripheral proprioceptive decline, although touch-induced illusion was also altered to a lesser extent [14, 15]. In addition, the influence of cerebral alterations with aging is supported by studies on artificial phantom limbs [59, 60], which demonstrate that the intention to move a paralyzed and anesthetized limb, that is, without afferent and motor fibers signal, results in an illusion of movement, suggesting that the perception of movement originates from the central rather than the peripheral pathway.

To our knowledge, only one study has investigated cerebral functional differences linked to VIM between young and older participants, showing that their cerebral patterns differ [16]. In fact, the VIM-induced activity was less lateralized in older people, whose resting-state connectivity between sensorimotor and frontoparietal regions was higher than that of younger people. These age-related structural and functional differences warrant further investigation regarding their relationship with discriminative ability and VIM strength.

### Does acute stroke exert an additional effect beyond aging on VIM discriminative ability and strength?

Since VIM was reduced in both older and acute stroke participants compared to young participants, and since vision did not modulate the VIM in the acute stroke group, aging appears to alter VIM response, with acute stroke exacerbating this impairment. This is further supported by a significant interaction effect between group and vision, suggesting that VIM becomes less distinct with age, a process exacerbated by acute stroke. This interpretation is reinforced by the significantly lower SKIP total scores observed in acute stroke participants compared to older participants without vision. Furthermore, only 11 out of the 26 (42%) acute stroke participants reported the VIM and, similarly, a prior study demonstrated that only 39% of participants with chronic stroke perceived the expected illusion, even when a virtual reality device was employed to facilitate the appearance of the illusion and its amplitude [30]. In our study, strokes involved right cortical and/or subcortical lesions (except in one individual) in the sensorimotor network. It has been suggested that older adults compensate their reduced VIM responses by increasing their sensorimotor and frontoparietal regions connectivity [16]. Thus, an impairment of this network could have further alter VIM responses. Furthermore, previous research suggested that frontoparietal cortical areas are involved in discriminative ability, while motor cortical areas play a role in VIM strength [18–24]. Moreover, the VIM is thought to be supported by a right frontoparietal dominance, with the parietal cortex contributing to motor intention and awareness [18, 19, 23, 61]. However, it is worth noting that when specifically evaluating VIM strength with participants who experienced the VIM, no significant differences could be shown between acute stroke participants and healthy participants. This results should still be interpreted with caution as unequal and small sample sizes were compared.

These mechanisms could be considered in rehabilitation settings, where leveraging the VIM could improve immediate motor task performance and prevent sensorimotor areas from functional alterations after limb immobilization [24, 28]. A recent study in hemiplegic subacute stroke patients highlighted significant improvements in hand motor performance following a two-week VIM training protocol [31]. The improvements, assessed with the Wolf Motor Function test, the 10-second test, wrist and finger motor items of the FMA, and the frequency of the Motor Activity Log amount of use, were sustained one month after training. Nevertheless, the authors failed to demonstrate a significant increase in µ-wave event-related desynchronization at the C3 and C4 loci. Yet, the long-term impact of VIM on motor and brain recovery remains uncertain. Future studies are needed to better characterize the factors shaping VIM responses in the stroke population and to optimize the timing of intervention, whether in the acute, subacute, or chronic phase.

Additionally, a strong correlation was observed between age and VIM amplitude in acute stroke participants independently of their clinical outcomes, suggesting a combined effect of aging and acute stroke sensorimotor network alteration. Not only does acute stroke impair VIM discriminative ability, but their interaction appears to eliminate VIM experience altogether. Thus, aging appears to be the primary factor influencing VIM responses in our study, with stroke compounding this decline.

In stroke rehabilitation, aging has been shown to be negatively correlated with functional outcomes, regardless of lesion severity [62]. This raises the possibility that age might interact with VIM responsiveness, ultimately shaping functional recovery. Clarifying this relationship could be valuable, as VIM might serve as a potential predictor of functional outcomes in stroke rehabilitation.

### Limitations

Even though we controlled for several confounding factors, unmeasured variables may still play a role. For instance, physical activity level could be an overlooked factor, as it may help to preserve the proprioceptive system with aging, particularly of the muscle spindle from which the VIM signal originates [63]. Additionally, personal education may have contributed, as it is believed to enhance cerebral reserve mechanisms throughout life [64], and both cortical and subcortical areas are involved in VIM responses. We also did not quantify cognitive abilities, notably in acute stoke participants, which could have influenced their understanding and responses. As the SKIP scale is subjective, it would have been valuable to compare our SKIP ordinal scale data with quantitative data obtained from inertial measurement units. This would have provided additional information about the occurrences of tonic vibration responses [43]. Finally, it is important to note a potential lack of statistical power with unequal and small sample sizes in the specific assessment of VIM strength between groups.

## Conclusion

To conclude, the present study demonstrated that the VIM can be effectively elicited in both young and older individuals. Nonetheless, the discriminative ability and VIM response were reduced in older participants, likely attributable to age-related changes in muscle spindles and central neural processing. In acute stroke participants, vision did not significantly modulate the VIM, suggesting an additional detrimental effect beyond aging. However, given the non-significant result, the absence of modulation cannot be firmly concluded and should be interpreted with caution. Taken together, the findings of this study suggest that aging was the primary factor that altered VIM responses, even among acute stroke individuals.

Given that physical activity and educational background are known to positively influence proprioceptive function and brain health in aging, further research is warranted to investigate their potential as a preventive measure to promote healthy aging through the measurement of VIM discriminative ability and strength.

## Supplementary Information

Below is the link to the electronic supplementary material.

Supplementary Material 1

## Data Availability

No datasets were generated or analysed during the current study.
