# Supplementary material for: Vibration-induced illusion of movement is hindered by acute stroke but mostly by aging: a cross-sectional study
Source: Aging Clin Exp Res. 2025 Dec 1;38(1):20. doi: 10.1007/s40520-025-03247-6 (PMC12775077; doi:10.1007/s40520-025-03247-6)
Supplement: Supplementary file 1 — Supplementary Material 1 [file 40520_2025_3247_MOESM1_ESM.docx]

**Supplementary materials**

Table 1: Functional localization of lesions in acute stroke patients

| **Patients** | **S1** | **M1** | **PMC** | **SMA** | **CSF** | **V1** |
| --- | --- | --- | --- | --- | --- | --- |
| 1 |  | X |  |  |  |  |
| 2 |  |  |  |  | X |  |
| 3 |  |  |  |  | X |  |
| 4 |  |  |  |  | X |  |
| 5 | X | X | X |  |  |  |
| 6 |  |  |  |  |  |  |
| 7 | X | X | X |  | X |  |
| 8 |  |  |  |  |  |  |
| 9 |  |  |  |  | X |  |
| 10 |  |  | X | X |  |  |
| 11 | X | X |  |  |  |  |
| 12 | X | X |  |  |  |  |
| 13 |  | X |  |  | X |  |
| 14 |  |  |  |  |  |  |
| 15 |  |  |  |  | X |  |
| 16 |  |  |  |  | X |  |
| 17 |  |  |  |  | X |  |
| 18 |  |  |  |  |  |  |
| 19 |  |  |  |  | X |  |
| 20 | X | X |  |  |  | X |
| 21 |  |  | X |  | X |  |
| 22 | X | X | X |  |  | X |
| 23 |  |  |  |  |  |  |
| 24 |  | X |  |  |  |  |
| 25 |  |  |  |  | X |  |
| 26 |  |  |  |  |  |  |
| ***CSF****: Corticospinal fasciculus;* ***M1****: Primary Motor cortex;* ***PMC****: Premotor Cortex;* ***S1****: Primary Somatosensory area;* ***SMA****: Supplementary Motor Area;* ***V1****: Primary visual cortex* | | | | | | |

Table 2: Anatomical localization of lesions in acute stroke patients

| **Patients** | **CN** | **LN** | **IC** | **EC** | **Th** | **CR** | **Ins** | **FC** | **PC** | **OC** | **P/Bstem** |
| --- | --- | --- | --- | --- | --- | --- | --- | --- | --- | --- | --- |
| 1 |  |  |  |  |  |  |  | X |  |  |  |
| 2 |  |  |  |  |  | X |  |  |  |  | X |
| 3 |  |  |  |  |  | X |  |  |  |  |  |
| 4 |  |  | X |  |  |  |  |  |  |  |  |
| 5 |  |  |  |  |  |  |  | X | X |  |  |
| 6 |  | X |  |  |  |  |  |  |  |  |  |
| 7 | X | X | X | X |  | X | X | X | X |  |  |
| 8 | X | X | X | X |  | X | X |  |  |  |  |
| 9 |  |  | X |  | X |  |  |  |  |  |  |
| 10 | X | X |  |  |  |  |  | X |  |  |  |
| 11 |  |  |  |  |  |  | X | X | X |  |  |
| 12 |  | X |  |  |  |  | X | X | X |  |  |
| 13 | X | X | X | X | X | X | X | X |  |  |  |
| 14 | X | X |  | X |  |  |  |  |  |  |  |
| 15 |  |  | X |  | X |  |  |  |  |  |  |
| 16 | X | X | X |  |  |  |  |  |  |  |  |
| 17 |  | X | X |  |  |  |  |  |  |  |  |
| 18 |  | X |  |  |  |  | X | X |  |  |  |
| 19 | X | X |  |  |  |  |  |  |  |  |  |
| 20 |  |  | X |  |  |  | X | X | X | X |  |
| 21 | X | X | X | X |  |  | X | X |  |  |  |
| 22 | X |  |  |  |  | X |  | X | X | X |  |
| 23 | X |  |  |  |  |  |  |  |  |  |  |
| 24 |  |  |  |  |  |  |  | X | X |  |  |
| 25 |  |  |  |  |  |  |  |  |  |  | X |
| 26 | X | X |  |  |  | X |  |  |  |  |  |
| ***CN****: Caudate Nucleus;* ***CR****: Corona Radiata;* ***EC****: External Capsule;* ***FC****: Frontal Cortex;* ***IC****: Internal Capsule;* ***Ins****: Insula;* ***LN****: Lenticular Nucleus;* ***P/Bstem****: Pons or Brain stem;* ***PC****: Parietal Cortex;* ***OC****: Occipital Cortex;* ***Th****: Thalamus* | | | | | | | | | | | |
